# Evolving understanding of cardiovascular, cerebrovascular and peripheral arterial disease in people living with HIV and role of novel biomarkers. A study of the Spanish CoRIS cohort, 2004-2015

**DOI:** 10.1371/journal.pone.0215507

**Published:** 2019-04-26

**Authors:** Mar Masiá, Sergio Padilla, José A. García, Javier García-Abellán, Marta Fernández, Ignacio Bernardino, Marta Montero, Joaquim Peraire, Berta Pernas, Félix Gutiérrez

**Affiliations:** 1 Infectious Diseases Unit, Hospital General Universitario de Elche and Universidad Miguel Hernández, Alicante, Spain; 2 Statistics, Centro de Investigación Operativa, Universidad Miguel Hernández, Elche, Alicante, Spain; 3 Infectious Diseases Unit, Hospital La Paz-Carlos III-Cantoblanco, Madrid, Spain; 4 Infectious Diseases Unit, Hospital La Fe, Valencia, Spain; 5 Infectious Diseases Unit, Hospital Universitari de Tarragona Joan XXIII, IISPV, Universitat Rovira i Virgili, Tarragona, Spain; 6 Infectious Diseses Unit, Complejo Hospitalario Universitario de A Coruña, A Coruña, Spain; Columbia University Medical Center, UNITED STATES

## Abstract

**Objectives:**

To analyze the incidence rates (IR) and spectrum of vascular events in people living with HIV (PLWH) in Spain from 2004 to 2015. Serial measurements of different plasma cardiovascular biomarkers were assessed in relation to disease development.

**Methods:**

Longitudinal study in a nationwide contemporary multicenter cohort of PLWH. A nested case-control study was performed to evaluate the predictive value of cardiovascular biomarkers. Additive generalized and Cox mixed models were used for the analyses.

**Results:**

9,712 PLWH and 48,341 person-years of follow-up were analysed. During 2004–2015, 147 persons developed 154 vascular events; 80 (54.42%) coronary-related; 65 (44.22%) cerebrovascular-related, and 9 (6.12%) peripheral arterial disease. The 2004–2015 IR (95% confidence interval) of vascular events was 3.17 (2.69–3.71) x1,000 person-years; 1.64 (1.30–2.05) for coronary events; 1.34 (1.03–1.70) for cerebrovascular events; and 0.19 (0.09–0.35) for peripheral arterial disease (p<0.001). IR of vascular events gradually increased from 0.37 (0.12–0.85) x1,000 patient-years in the stratum 25-34-years to 19.65 (6.38–45.85) x1,000 patient-years in the stratum 75-84-years. Compared to the general population, there was a higher incidence of acute myocardial infarction (AMI) in men (sIR ratio 1.29 [95% CI 1.16–1.42]), of cerebrovascular events in women (sIR ratio 2.44 [95% CI 1.68–3.19]), and of both types of events specifically among the younger age-strata. CD4 count (hazard ratio 0.80, [95% CI, 0.79–0.81]), age (1.86 [1.47–2.34] for 45–65 years and 3.44 [2.37–4.97] for >65 years) and vascular event (1.81 [1.12–2.94]) were associated with total mortality. Adjusted levels of intercellular-adhesion-molecule (sICAM), pro-b-type-natriuretic-peptide (pro-BNP) and marginally sCD14, were higher among patients who subsequently developed vascular events.

**Conclusion:**

Vascular events in PLWH do preferentially occur in the older age-strata, they are associated with increased mortality and, compared to the general population, the excess risk occurs at younger ages. Peripheral arterial disease is unusual. Vascular events are preceded by increased levels of sICAM, pro-BNP and, marginally, sCD14.

## Introduction

The increase in life expectancy associated with effective antiretroviral therapy (ART) and the predominance of non-AIDS over AIDS events, has led physicians to focus attention of people living with HIV (PLWH) on common health problems that also affect the HIV-negative population. Cardiovascular disease is one of the leading causes of morbidity and mortality in PLWH [1]. Coronary and cerebrovascular events have been described to occur more frequently among HIV-infected persons than in their HIV-negative pairs [2,3], with most of the available data coming from Northern European countries and the USA, where the incidence of cardiovascular disease is relatively high.

Several factors have been implicated in the pathogenesis of enhanced atherosclerosis in PLWH, although it remains to be completely elucidated. In addition to the traditional cardiovascular risk factors, variables intrinsically linked with HIV infection, like ART, and also inflammation and immune activation, mainly in association with uncontrolled viremia and low CD4 cells, seem to play a central role [4–6]. To predict the risk of developing cardiovascular disease in an individual person with HIV, different risk scores mainly based on traditional cardiovascular risk factors have been used, including equations specifically adapted to the HIV population, as well as inflammation and immune activation biomarkers. The cardiovascular scores have not generally shown an optimal ability to predict atherosclerotic disease in PLWH [7]. The experience with the biomarkers is still scarce, and most of the available data include isolated baseline measurements of some biomarkers like highly sensitive C-reactive protein (hsCRP), interleukin-6 (IL-6) and D-dimer as predictors of cardiovascular disease development [6], but not serial determinations under different conditions, including after ART initiation, have yet been analyzed.

Spain has a low incidence of atherosclerotic disease in the general unselected population [8], but no data are yet available on the frequency and spectrum in the HIV population. We aimed to describe the frequency of coronary, cerebrovascular and peripheral arterial disease (PAD) in a contemporary multicenter cohort of PLWH in Spain from 2004 to 2015. We also assessed longitudinal measurements of several cardiovascular biomarkers in relation to the development of vascular events.

## Methods

The Cohort of adult PLWH of the AIDS Research Network (CoRIS) is an open, prospective, multicentre cohort of adult subjects with confirmed HIV infection, naïve to ART at study entry, who are recruited in HIV care units of the Spanish Public Health System [9], which constitutes the standard place of treatment for the great majority of persons in Spain. CoRIS was launched in 2004. Each centre recruits into the cohort all subjects seen for the first time in the centre who meet the following criteria: over 13 years of age, confirmed HIV diagnosis, and naive to ART. Written informed consent is obtained from all the patients. Demographic, clinical, laboratory, microbiological and treatment information is recorded. For these analyses, patients’ follow-up data were administratively censored on December 31, 2015.

The cohort is linked to a centralized BioBank, where patients’ blood samples are processed, cryopreserved immediately after reception and stored [10]. Participating centres are encouraged to obtain a first blood sample at engagement in the cohort, preferentially before starting ART, and follow-up samples preferentially annually, or at least biannually thereafter. The BioBank has obtained the UNE-EN-ISO 9001:2008 Systems of Quality Management Requirements. Approval from each hospital’s Ethics Committee, including the Ethics and Clinical Research Committee of Hospital General de Elche, Hospital Ramón y Cajal, Hospital Universitario Donostia, Hospital Universitario de Canarias, Santa Cruz de Tenerife, Hospital de Navarra, Pamplona, Hospital Universitario Joan XIII, Tarragona, and Hospital Universitario Son Espases, Palma de Mallorca, and written informed consents from the patients, including the specific consent for the BioBank, were obtained.

### Non-AIDS events classification

All centres were invited in February 2008 to provide a pre-specified list of ischemic vascular diseases, and other incident NAEs (S1 File). Cardiovascular events included acute myocardial infarction (AMI), angina, congestive heart failure and coronary-related death; cerebrovascular events included stroke and transient ischemic attack (TIA). Definition criteria for each event are detailed in S1 File. For cardiovascular diseases, definition criteria were based on the DAD case definitions [11]. Briefly, definitive AMI was established with a diagnostic electrocardiogram (ECG), or the presence of symptoms + probable ECG + rise of cardiac biomarkers or the presence of typical symptoms + cardiac biomarkers elevation+ not codifiable or not available ECG with signs of ischemia. No cases of probable AMI were included. Angina was defined as symptoms suggestive of myocardial ischemia, such as thoracic pain, or pain in the jaw or the arm, with changes in ECG that confirm the existence of myocardial ischemia, such a depression of at least 0.5 mm of ST segment or T wave reversal of at least 1 mm in 2 or more contiguous derivations. Sudden death of possible coronary etiology considered as typical, atypical or not enough described symptoms and previous history of coronary disease or evidence of coronary disease on autopsy. Stroke was defined as a neurological deficit which does not change during the first 24–72 hours after the initiation. TIA, as focal neurological deficit due to ischemia of a cerebral territory that lasts less than 24 hours (S1 File). PAD was defined as abnormal ankle-brachial index (<0.9), intermittent claudication or any other clinical finding including arterial revascularization or previous amputation.

Centres were asked to collect retrospectively all of the above NAEs occurring from the day of entry in the cohort to February 2008, and to report them prospectively since then. They were provided a structured event reporting form containing the list of events to be reported, and the precise definition of each NAE required for the inclusion [12]. To guarantee that no underreporting had occurred, we cross-checked the CoRIS cohort database with the national hospital discharges for AMI or cerebrovascular events with HIV infection as a secondary diagnosis obtained from the Minimum Basic Data Set (CMBD) that all Spanish hospitals have reported yearly to the Health Ministry since 1990. Available variables to cross-check between the two registries were sex, birth date and the autonomous community. If non-reported vascular events were found, each centre was contacted in January 2017 and asked to check if they were indeed non-notified cases of vascular events, and to report them if they were confirmed as new cases.

### Plasma biomarkers

Blood samples for the plasma cardiovascular biomarkers analyses were kindly provided by the BioBank. Plasma aliquots obtained were stored at -80°C. All frozen samples were subsequently defrosted for their analysis. We selected a panel of biomarkers of inflammation, thrombosis and vascular function which have been extensively analyzed in the HIV population as predictors of cardiovascular disease [13]. Commercially available ELISA kits were used to measure plasma levels of vascular cell adhesion molecule-1 (sVCAM-1), intercellular CAM-1 (sICAM-1) and sCD14 (Quantikine, R&D Systems Europe Ltd, UK), interleukin-6 (IL-6), tumour necrosis factor-alpha (TNF-α), sCD40 and sCD163 (DuoSet, R&D Systems Europe Ltd, UK), D-dimer (Technozym D-Dimer ELISA, Technoclone GmbH, Vienna/Austria), neopterin (IBL International GmbH, Hamburg/Germany) and 8-isoprostane (Cayman Chemical Company, Michigan/USA). Highly-sensitive C-reactive protein (hsCRP) was measured with a chemiluminescent immunometric assay (Immulite 2000 autoanalyzer, Siemens, Germany). Malondialdehyde (MDA) was measured with a commercial high performance liquid chromatography (HPLC) kit (Chromsystems Instruments & Chemicals GmbH, Gräfelfing/Germany). N-terminal pro b-type natriuretic peptide (NT-ProBNP) was measured with a chemiluminescent immunometric assay (VITROS 5600 integrated system, Ortho-Clinical Diagnostics, USA).

### Statistical analyses

Descriptive analyses were used to summarize the incidence rates of vascular events, and baseline characteristics of the patients developing them were compared with the patients without vascular events from the cohort with the Mann-Whitney or Chi square and Fisher’ exact test where applicable. The association of the patients’ characteristics with the development of vascular events was adjusted with a Cox mixed-effect model including the significant factors found in the univariate analysis. An additional adjusted analysis was carried out including the cardiovascular risk factors (hypertension, smoking habit, and total cholesterol) in the subgroup of patients for whom the information was available. Incidence rates of clinical events were calculated as the number of new events divided by the number of person-years of follow-up. Follow-up for the analysis of incident NAEs accrued from the day of inclusion in the cohort to the date of the development of a NAE. Only first vascular events of each category (coronary, cerebrovascular and peripheral arterial) occurring from the day of cohort entry were included in analyses. Follow-up of patients not developing events accrued from the day of inclusion in the cohort to the date of last visit or death. Data were abstracted from the study database (S2 File) on December 2015.

To calculate the IR of AMI and cerebrovascular disease in the general population in Spain, the national rates of hospital discharges for AMI and cerebrovascular disease per 100.000 habitants stratified for age and sex from 2004 to 2015 were analyzed using the morbidity report data from the National Statistics Institute [14]. Age standardized IR of AMI and cerebrovascular disease in PLWH from CoRIS were compared with the IR of AMI and cerebrovascular disease in the general Spanish population. Standardization was performed with the direct method using the European population as reference. For the comparison between infected and uninfected populations, analyses included the first and recurrent events occurring during follow-up.

To analyse the contribution of atherosclerotic events to total mortality, a Cox mixed effects model was run adjusted for age (categorized into 3 groups: <45 years; 45–65 years; and 65 years), sex, serial CD4 cells measures and HIV RNA at cohort entry.

The relationship of plasma cardiovascular biomarkers with the development of vascular events was assessed with a nested case-control study, including all patients with vascular events and a blood sample available at the Biobank and two age and sex-matched randomly selected controls that entered the cohort during the same period and did not develop atherosclerotic events. Baseline and all available blood samples after virological suppression and before event development were used for analysis. Censoring date for this sub-study was October 31, 2010. Additive generalized models were used to analyze the relationship of serial measurements of plasma cardiovascular biomarkers with the development of vascular events. Models were adjusted for age, sex, CD4 cell count and HIV RNA at cohort entry, transmission risk group and hepatitis C viral infection.

## Results

Overall, 9,712 PLWH and 48,341 person-years of follow-up were analysed. Demographic and clinical characteristics of the participants are provided in Table 1. From 2004 to 2015, 147 persons developed 154 vascular events; 80 (54.42%) coronary-related events, 65 (44.22%) cerebrovascular-related events, and 9 (6.12%) PAD. Coronary events included AMI in 58 (72.50%) participants, angina in 17 (21.25%), and sudden death of probable coronary etiology in 5 (6.25%). Cerebrovascular disease included ischemic stroke/TIA in 54 (83.08%) participants, and haemorrhagic stroke in 11 (16.92%) (Table 1). Twenty-four (16.32%) of the persons developing vascular events died. Of them, 7 cases were attributed to cerebrovascular disease, 2 to coronary/heart disease and 15 to other causes (Table 1).

**Table 1:** Baseline characteristics of the patients. Numbers in parenthesis represent percentages for categorical variables, or first and third quartiles for continuous variables. IDU, intravenous drug user; MSM, men who have sex with men; TIA, transient ischemic attack.

Variable	Patients developing vascular events N = 147	Patients not developing vascular events N = 9,566	p
Sex, male	123 (83.7)	7,863 (82.2)	0.851
Age at inclusion in the Cohort	47.5 (41–55)	36 (29–43)	<0.001
HIV transmission risk group			<0.001
IDU	13 (8.8)	948 (9.9)	
Sexually transmitted	123 (83.7)	8,232 (85.8)	
Other	3 (2.0)	3 (0.03)	
Unknown	8 (5.4)	8 (0.1)	
Country of origin			
Spain	120 (81.6)	6,792 (71)	0.003
Other	26 (2)	2,774 (29)	
Level of education			0.106
No studies	8 (7)	392 (4)	
Primary	16 (11)	710 (7)	
High school	48 (32)	2,230 (23.3)	
Bachelor	25 (19)	2,388 (25)	
University	27 (18.2)	2,108 (22)	
Other	8 (2)	158 (1.7)	
Unknown	18 (10)	1,580 (17)	
Hepatitis C virus infection			
Positive	101 (17.7)	1,347 (17.2)	0.550
Negative	24 (82.4)	6,478 (82.8)	
AIDS diagnosis	44 (30)	1,602 (17)	<0.001
CD4 at cohort entry	240 (70–487)	370 (193–560)	<0.001
Nadir CD4	157 (44–318)	274 (145–399)	<0.001
HIV RNA at cohort entry	100,000 (22,547–292,052)	48,800.0 (11,500–154.794)	0.016
Smoking habit (N = 4,798)	30 (66.7)	2349 (49.4)	0.024
Hypertension (N = 1,966)	26 (70.2)	827 (42.9)	0.001
Total cholesterol, mg/dl (N = 7,661)	199	173.2	<0.001
Mortality	24 (16.3)*	102 (1.07)	<0.001
Vascular events	154		
Coronary events	80 (54.4)		
Acute myocardial infarction	58 (39.5)		
Angina	17 (11.6)		
Death of probable coronary etiology	5 (3.4)		
Cerebrovascular events	65 (44.2)		
Ischemic stroke/TIA	54 (36.7)		
Haemorrhagic stroke	11 (7.5)		
Peripheral arterial disease	9 (6.1)		

*7 cases were attributed cerebrovascular disease, 2 to coronary/heart disease and 15 to other causes

Participants developing vascular events were older at cohort inclusion (median [interquartile range, IQR] 47.5 [41–55] years vs 36 [29–43] years, p<0.001), the transmission risk group was more frequently heterosexual and less frequently men who have sex with men (p<0.001), had been more frequently diagnosed with AIDS (<0.001), and had lower CD4 cell count nadir (p<0.001), lower CD4 cell count at cohort entry (p<0.001) and higher HIV RNA (p = 0.016) (Table 1). Mortality was also higher in persons with vascular events (p<0.001). In a multivariate Cox mixed-effect model, the factors independently associated with vascular events were age (HR 1.08 per age year increase, [95% CI, 1.07–1.10], p<0.001), CD4 cell nadir (HR 0.93 [95% CI, 0.90–0.97] per squared-root CD4 cell/μL increase, p = 0.001), and heterosexual HIV transmission (HR 1.73 [95% CI, 1.16–2.59], p = 0.007). In an analysis restricted to participants for whom information on cardiovascular risk factors was available (see Table 1), vascular events were more frequent among older participants, smokers, subjects with heterosexual HIV transmission, and among participants with higher levels of systolic blood pressure, total serum cholesterol and serum creatinine. In a multivariate analysis within this last subgroup, the independent factors associated with vascular events were age (HR 1.09 [95% CI, 1.06–1.13] per age year increase, p<0.001), CD4 cell nadir (HR 0.91 [95% CI, 0.84–0.98] per squared-root CD4 cell/μL increase, p = 0.018), smoking habit (HR 4.51 [95% CI, 1.96–10.42], p = 0.001), total cholesterol (HR 1.01 [95% CI, 1.00–1.02], p = 0.045) and systolic blood pressure (HR 1.00 [95% CI, 0.99–1.00], p = 0.042). In a sensitivity adjusted analysis including only participants who developed coronary events, age (HR 1.08 [95% CI, 1.02–1.13] per age year increase, p = 0.001), heterosexual risk of HIV acquisition (HR 3.50 [95% CI, 1.12–10.94], p = 0.030), CD4 cell nadir (HR 0.89 [95% CI, 0.79–0.99, p = 0.038) per squared-root CD4 cell/μL increase, total serum cholesterol levels (HR 1.02 [95% CI, 1.01–1.03] per mg/dL increase; p = 0.0007) and smoking (HR 12.85 [95% CI, 2.58–64.01], p = 0.001) were associated with event development. In a sensitivity adjusted analysis including only participants who developed cerebrovascular events, older age (HR 1.13 [95% CI, 1.07–1.18] per age year increase, p <0.001), systolic blood pressure (HR 1.00 [95% CI, 1.00–1.01], p = 0.034) and female sex (HR 4.68 [95% CI, 1.20–18.24]; p = 0.026) were found to be associated with CVEs.

**Fig 1:**
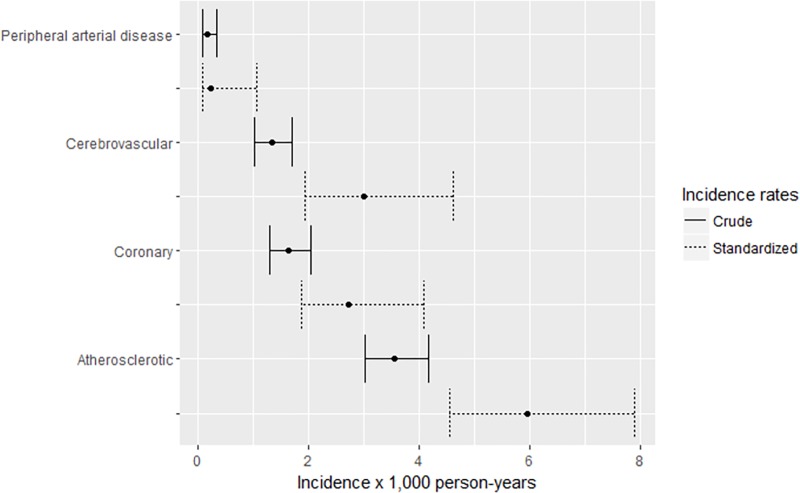
Crude and standardized incidence rates of vascular events per 1,000 person-years by event category, 2004–2015.

The 2004–2015 crude incidence rate (IR) (95% confidence interval, CI) of vascular events was 3.17 (2.69–3.71) per 1,000 person-years; 1.64 (1.3–2.05) per 1,000 person-years for coronary events; 1.34 (1.03–1.70) per 1,000 person-years for cerebrovascular events [1.12 (0.84–146) per 1,000 person-years for ischemic stroke/TIA and 0.22 (0.11–0.41) per 1,000 person-years for haemorrhagic stroke]; and 0.19 (0.09–0.35) per 1,000 person-years for peripheral arterial disease (p<0.001). Crude and standardized incidence rates (sIR) of all vascular events, and by event category are shown in Fig 1.

**Fig 2:**
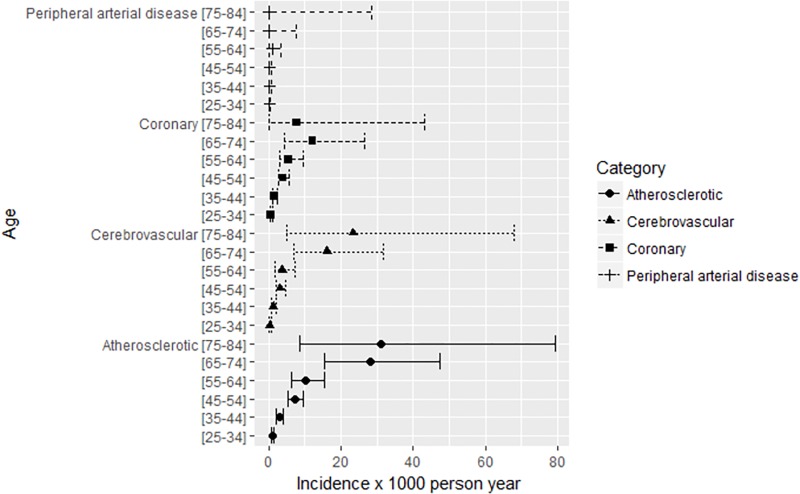
Incidence rates of vascular events by category and by age group, 2004–2015.

The sIRs of vascular events increased after the standardization with the European general population probably due to the lower age of patients included in the CoRIS cohort. Fig 2 shows incidence rates of coronary, cerebrovascular and peripheral arterial disease by age strata.

There was an expected increase in the IR of vascular events with increasing age, from 0.37 (0.12–0.85) x1,000 patient-years in the age stratum 25–34 to 19.65 (6.38–45.85) x1,000 patient-years in the stratum 75–84. Confidence intervals widened from age-strata above 55 years due to the lower number of patients included.

The contribution of vascular events to mortality was analysed with a Cox mixed effects model. The analysis showed that CD4 cell count had a protective effect on mortality (hazard ratio [HR] 0.80, 95% CI 0.79–0.81; p<0.001) and age (HR 1.86 [1.47–2.34] for the 45–65 years stratum and 3.44 [2.37–4.97] for >65 years stratum, p<0.001 for both) and having had a vascular event (HR 1.81 [1.12–2.94]; p = 0.015) were associated with increased risk for mortality.

The age-standardized IR (sIR) of events requiring hospital admission in PLWH were compared with those occurring in the general Spanish population with the morbidity report data from the National Statistics Institute (INE). Table 2 shows the incidence rates and rate ratios of AMI and cerebrovascular events per 1,000 person-years by sex and age strata in CoRIS participants and in the general population. The sIR of AMI in CoRIS from 2004 to 2015 was 2.38 (95% CI: 2.26–2.50) per 1,000 person-years in men, and 0.66 (95% CI: 0.17–3.21) per 1,000 patient-years in women. The sIR of AMI during the same period in the male uninfected population was 1.85 (95% CI: 1.75–1.95) per 1,000 person-years in men and 0.39 (0.39–0.40) per 1,000 person-years in women. The IR ratio of AMI in males was 1.29 (95% CI 1.16–1.42, P = 0.045) (Table 2). By age strata, the relative risk of AMI was higher among the four younger 10-year groups from 25 to 64 years in the analysis including both sexes, and in the 25–34 and 45–54 year strata in men. The sIRs (95% CI) per 1,000 person-years in participants from CoRIS of any CVE, i-STR/TIA and h-STR in PLWH from 2004 to 2015 were 2.60 (1.84–3.64), 2.30 (1.58–3.29) and 0.30 (0.10–0.82), respectively. Compared to the general population, there was a 42% higher incidence [sIR ratio (95%CI) 1.42 (1.10–1.74), p = 0.038] for CVE, 47% higher incidence [sIR ratio (95%CI) 1.47 (1.05–2.08), p = 0.032] for i-STR/TIA, and similar incidences for h-STR [sIR ratio (95%CI) 1.10 (0.47–2.59), p = 0.388]. When analysed by gender (Table 2), the higher incidence in PLWH was seen in women [3.24 (1.27–8.20) per 1,000 person-years in PLWH vs 1.33 (1.32–1.33) per 100,000 person-years in the GP; sIR ratio (95%CI) 2.44 (1.68–3.19), p = 0.027] while not in men [2.44 (1.63–3.57) in PLWH vs 2.38 (2.38–2.39) in GP; sIR ratio (95%CI) 1.02 (0.65–1.38), p = 0.396]. In the analysis including both sex groups, CVE sIRs in PLWH remained above sIRs in the general population in all age strata (25–34, 35–44, 45–54, 55–64, 65–74 and 75–84), and were significantly higher in the 35–44 [sIR ratio (95%CI) 2.44 (1.39–3.97), p = 0.003] and the 45–54 [sIR ratio (95%CI) 1.91 (1.25–2.84), p = 0.005] year age strata. Similar results were found for the i-STR/TIA [sIR ratio (95%CI) 2.05 (1.02–3.68), p = 0.042 for the 35–44 age strata; 1.89 (1.16–2.93), p = 0.012 for the 45–54 age strata], and only in the 35–44 age strata [sIR ratio (95%CI) 4.14 (1.34–9.67), p = 0.016] for h-STR.

**Table 2:** Crude Incidence rates per 1,000 person-years of acute myocardial infarction and cerebrovascular events in PLWH by sex and age stratum in the Cohort of HIV Adults of the AIDS Research Network (CoRIS) compared to the Spanish general population.

	General population		CoRIS				
Age stratum (years)	IR	(95% CI)	IR	(95% CI)	IRR	(95% CI)	p value
Cerebrovascular events							
Both sexes							
25–34	0.12	(0.12–0.12)	0.21	(0.4–0.64)	1.74	(0.36–5.11)	0.493
35–44	0.37	(0.37–0.37)	0.91	(0.52–1.48)	2.44	(1.39–3.97)	0.003
45–54	1.15	(1.15–1.16)	2.21	(1.41–3.29)	1.91	(1.22–2.84)	0.005
55–64	2.85	(2.84–2.87)	3.20	(1.59–5.72)	1.12	(0.55–2.00)	0.788
65–74	6.60	(6.57–6.62)	9.31	(4.25–17.68)	1.41	(0.64–2.67)	0.388
75–85	12.96	(12.92–12.99)	15.71	(4.28–40.24)	1.21	(0.33–3.10)	0.839
All ages*	1.83	(1.82–1.83)	2.60	(1.84–3.64)	1.42	(1.10–1.74)	0.038
Males							
25–34	0.12	(0.12–0.13)	0.18	(0.02–0.65)	1.41	(0.17–5.10)	0.827
35–44	0.43	(0.42–0.44)	0.84	(0.43–1.48)	1.95	(1.00–3.40)	0.048
45–54	1.53	(1.51–1.54)	1.71	(0.96–2.83)	1.12	(0.62–1.85)	0.721
55–64	4.03	(4.01–4.05)	2.51	(1.01–5.18)	0.64	(0.25–1.28)	0.259
65–74	8.77	(8.73–8.81)	10.84	(4.96–20.59)	1.23	(0.56–2.34)	0.616
75–85	15.31	(15.25–15.37)	13.14	(2.71–38.41)	0.85	(0.17–2.50)	1.000
All ages*	2.38	(2.38–2.39)	2.44	(1.63–3.57)	1.02	(0.65–1.38)	0.396
Females							
25–34	0.12	(0.11–0.12)	0.37	(0.01–2.10)	3.08	(0.07–17)	0.553
35–44	0.31	(0.30–0.31)	1.20	(0.32–3.08)	3.85	(1.05–9.8)	0.043
45–54	0.78	(0.77–0.79)	3.75	(1.62–7.40)	4.78	(2.06–9.4)	0.001
55–64	1.73	(1.71–1.74)	6.10	(1.66–15.63)	3.52	(0.96–9.0)	0.057
65–74	4.71	(4.68–4.73)	0	(0–26.97)	0	(0–5.7)	1.000
75–85	11.31	(11.27–11.36)	32.02	(0.80–178.41)	2.82	(0.07–15.7)	0.595
All ages*	1.33	(1.32–1.33)	3.24	(1.27–8.20)	2.44	(1.68–3.1)	0.027
Acute myocardial infarction							
Both sexes							
25–34	0.04	(0.03–0.04)	0.35	(0.11–0.82)	8.91	(3.7–21.4)	0.001
35–44	0.30	(0.30–0.30)	0.72	(0.37–1.26)	2.37	(1.35–4.1)	0.002
45–54	1.07	(1.07–1.08)	2.71	(1.75–4.00)	2.51	(1.7–3.72)	0.001
55–64	1.93	(1.92–1.94)	3.61	(1.73–6.65)	1.87	(1.01–3.47)	0.044
65–74	2.98	(2.97–3.00)	5.54	(1.51–14.19)	1.86	(0.7–4.95)	0.209
75–85	3.64	(3.63–3.66)	5.38	(0.13–29.97)	1.47	(0.21–10.47)	0.696
All ages*	1.00	(1.00–1.01)	2.10	(1.42–3.13)	2.08	(1.73–2.44)	0.001
Males							
25–34	0.06	(0.06–0.07)	0.34	(0.09–0.88)	5.11	(1.92–13)	0.001
35–44	0.52	(0.51–0.52)	0.80	(0.40–1.43)	1.54	(0.85–2.7)	0.148
45–54	1.86	(1.85–1.88)	2.90	(1.81–4.39)	1.55	(1.02–2.3)	0.037
55–64	3.29	(3.27–3.31)	4.39	(2.10–8.08)	1.33	(0.72–2.4)	0.361
65–74	4.63	(4.60–4.66)	6.50	(1.77–16.64)	1.40	(0.53–3.7)	0.497
75–85	6.75	(6.71-.79)	5.65	(0.14–31.52)	0.84	(0.12–5.9)	0.859
All ages*	1.85	(1.75–1.95)	2.38	(2.26–2.50)	1.29	(1.16–1.42)	0.045
Females							
25–34	0.01	(0.01–0.02)	0.40	(0.01–2.27)	36.7	(5–261)	0.001
35–44	0.08	(0.08–0.09)	0.35	(0.08–1.96)	4.28	(0.6–30.4)	0.113
45–54	0.29	(0.28–0.29)	1.82	(0.37–5.34)	6.3	(2–19.5)	0.001
55–64	0.63	(0.62–0.64)	0	(0–7.52)	0	-	-
65–74	1.55	(1.53–1.56)	0	(0–34.76)	0	-	-
75–85	2.20	(2.18–2.22)	0	(0–403.15)	0	-	-
All ages*	0.39	(0.39–0.40)	0.66	(0.17–10.21)	1.68	(0.67–2.69)	0.178

IRR, Incidence Rate Ratio.

*The standardized IRR was provided in this summarized category.

### Biomarkers levels

Baseline and follow-up plasma levels of different inflammation and immune activation (hsCRP, Il-6, TNF- α, ICAM, VCAM, neopterin, sCD14, sCD40, sCD163, MDA, isoprostane), coagulation (D-dimer) and myocardial function biomarkers (pro-BNP) were determined in 25 cases with vascular events and 52 controls without events (Table 3).

**Table 3:** Absolute difference in the levels of the biomarkers between participants who developed vascular events and patients who did not.

	Unadjusted analysis		Adjusted analysis#	
Biomarker	Absolute difference (standard error) in biomarkers levels*	p	Absolute difference between biomarkers (standard error)	p
**hsC-reactive protein (mg/dL)**	+6.58 (4.84)	0.178	+8.13 (5.31)	0.130
**TNF-alpha**	-78.11 (71.50)	0.278	-110.08 (79.41)	0.170
**IL-6 (pg/mL)**	-6.95 (8.13)	0.395	-9.96 (9.06)	0.276
**Neopterin (nmol/L)**	+4.62 (4.13)	0.267	+6.80 (4.19)	0.109
**s-VCAM**	-242.3 (186.1)	0.195	-116.2 (186.1)	0.534
**s-ICAM**	+58.29 (81.43)	0.476	+187.69 (81.99)	0.025
**D-dimer (ng/mL)**	-72.99 (71.89)	0.313	+9.37 (80.50)	0.907
**Free-F2-isoprostanes (pg/mL)**	-23.96 (28.53)	0.403	-27.19 (32.10)	0.400
**Malondialdehyde**	+0.50 (1.19)	0.677	-0.01 (1.32)	0.996
**s-CD14 (pg/mL)**	+545.9 (375.8)	0.150	+703.42 (386.62)	0.073
**s-CD163 (ng/mL)**	-96.01 (136.03)	0.483	-137.1 (150.7)	0.366
**s-CD40 (pg/mL)**	+72.23 (115.13)	0.532	75.83 (127.16)	0.553
**BNP**	+158.15 (76.59)	0.042	+196.64 (84.63)	0.023

*Participants with vascular events (n = 25) vs participants with no vascular events (n = 52).

^#^Adjusted for age, sex, HIV transmission category, hepatitis C infection, CD4 cell count and HIV RNA. Median (Q1,Q3) number of samples per patient was 1 (1–2.75). In the adjusted analysis, the relationship of each biomarker with vascular events was analysed separately, without the inclusion of the other biomarkers.

Median (range) number of determinations was 2 (1–3) per patient. After adjustment for age, sex, baseline CD4 count and HIV-RNA, HIV transmission group and hepatitis C coinfection, only the levels of sICAM (P = 0.025) and pro-BNP (P = 0.023) were found to be higher among persons who subsequently developed cardiovascular events, and there was a marginal association with sCD14 levels (P = 0.073), compared with persons without vascular events (Table 3).

## Discussion

This study represents the first nationwide cohort study analyzing the incidence and spectrum of coronary, cerebrovascular and peripheral arterial disease in PLWH in Spain. CoRIS is a contemporary cohort that recruits patients at 28 sites from 13 of the 17 Autonomous Communities in Spain, and it covers most of the country, specifically those areas with the highest HIV/AIDS prevalence according to national surveillance [9]. Incident vascular events were collected from 2004 to 2015. In a previous cohort analysis, cardiovascular events constituted the fifth most common cause of non-AIDS events after psychiatric, hepatic, malignant and renal events [12]. As expected, the incidence of cardiovascular and cerebrovascular disease in the Spanish PLWH patients was lower compared to the rates described in North America and Northern Europe [2,4,15–17]. We found a similar incidence rate of AMI to that described in France [18]. Lifestyle habits, particularly the diet, and probably genetic factors seem to play a crucial role in the low rates of cardiovascular disease found in Mediterranean countries, despite high prevalence of cardiovascular risk factors, like smoking habit [19]. The lower frequency of atherosclerotic disease observed in our study is in agreement with the small number of PLWH with high estimated cardiovascular risk using different scores described in a subgroup of participants from CoRIS, even though the prevalence of smokers was 46% [7].

Despite the relatively lower frequency of vascular events in our cohort compared with other Western countries, the incidence rates of both AMI and cerebrovascular events were higher than in the general Spanish population. In addition to the higher prevalence of cardiovascular risk factors described in PLWH [4], coronary and cerebrovascular events have been associated with HIV-associated factors, like low nadir and low recent CD4 cell counts [20,21], higher HIV replication [22], and with exposure to certain antiretroviral drugs, including lopinavir/ritonavir, indinavir, ddI and abacavir [23,24]. In fact, when we adjusted for traditional cardiovascular risk factors in the subgroup of patients for whom the information was available, the nadir CD4 cell count and the HIV-RNA turned out to be predictors of vascular events. The increase described in our cohort in the proportion of blood samples with CD4 cell counts >500 cells/μL and HIV RNA <50 c/ml, and the decrease in the use of protease inhibitors [25] might help reducing the incidence of ischemic events within the HIV population. Indeed, we have recently reported a reduction in the IR of AMI within this cohort although, despite this reduction, the IR was still higher than in the general Spanish population [26]. Our results should encourage physicians to make more intense efforts to achieve an optimal control of cardiovascular risk factors in addition to improving the virological and immunological control of PLWH to aim at attaining comparable incidence rates as in the general population. It should be stated that CoRIS in an open cohort, which contributes to explain the high proportion of blood samples with detectable HIV RNA levels and with CD4 cell counts lower than 500 cells/μL, since an entry criterion is being naïve to ART. The new recommendations endorsed in the guidelines regarding the moment of ART initiation might hopefully help decreasing both percentages in the near future, and contribute to reduce atherosclerotic disease.

Our study showed that the rates of vascular events increased with age, with a majority of events occurring within the older age strata. However, we found that the excess risk compared to the general population occurred mainly in the younger age strata in the CoRIS participants. This is congruent with data reporting younger mean age at presentation in the HIV population [27], and supports that, in addition to the older PLWH, preventive efforts should also be considered in the younger age groups, who usually have lower scores in the cardiovascular risk equations. Interestingly, such equations have shown to under-predict cardiovascular events just among the low-risk individuals (<10% 10-year atherosclerotic cardiovascular disease) [28]. This reinforces the urgent need to develop alternative cardiovascular risk scores for the HIV population capable of identifying these subjects as high-risk candidates for the adoption of preventive measures. We also found that women with HIV were more prone to have CVE than their HIV-negative pairs, while this difference was not observed with men, even though the absolute IR in CoRIS was lower in women. Different causes have been implicated for this gender-dependent increased risk, such as a higher prevalence of atherosclerotic plaques in women with HIV [29], less monitoring of interventions to prevent cardiovascular risk in women with HIV [30], and increased immune activation associated with female gender [31]. The relevance of prevention is emphasized by the fact that mortality was twice-as-high in PLWH with vascular events compared to those without events. Such results support data recently reported in our cohort, where non-AIDS events occurrence, no matter the degree of severity, was associated with increased risk for mortality [32].

The rates of cardiovascular and cerebrovascular events were similar in our cohort. These results differ other cohorts, where the frequency of cardiovascular morbidity usually exceed that of the cerebrovascular [32,33]. The lower frequency of cardiovascular disease in Spain, the better control of cardiovascular risk factors, which might preferentially have an impact on cardiovascular events, and the increasing age of PLWH might be factors involved in the higher current frequency of cerebrovascular disease observed in our cohort. In contrast, the incidence of PAD was much lower. This finding has also been reported in population-based studies performed in the general population in Spain [34]. PAD is associated with older age, diabetes, smoking and low CD4 cell counts [35]. These results are in agreement with the low frequency of the other clinical manifestations of the spectrum of atherosclerotic disease found in this cohort, probably because of the differences in HIV-related factors compared to older cohorts, and the beneficial contribution of genetic factors. Although high frequencies of PAD have been described in older HIV-infected populations [35], the low incidence in our cohort corroborates the results of previous studies reporting a low prevalence of PAD in PLWH [36,37], which was found to be comparable to that of the general population [38].

Longitudinal analyses of the biomarkers sICAM and pro-BNP showed higher levels in persons subsequently developing cardiovascular events after adjusting for demographic and HIV-associated factors. To the best of our knowledge, this is the first study to assess the role of repeated measurements of cardiovascular disease biomarkers as predictors of events development, although the unavailability of cardiovascular risk factors for adjustment limits the predictive value of the results obtained. Amino-terminal pro-B-type natriuretic peptide (NT-proBNP) is a cardiac hormone secreted by cardiomyocytes in response to an increase in ventricular wall stretch. In addition to being a marker of cardiac dysfunction, increased circulating levels of NT-proBNP are predictors of coronary heart disease and stroke in the general population [39]. The association of increased levels of pro-BNP with cardiovascular events occurrence in PLWH had only been shown in the SMART trial and the HIV-HEART study, in both cases with baseline pro-BNP levels [40, 41]. Availability of biomarkers levels during follow-up allowed assessing their predictive role also in patients receiving ART, and at a nearest time to event development. ICAM is part of the immunoglobulin superfamily, which plays an important role on different stages of atherosclerosis, including inflammation, immune response and intracellular signaling event [42]. In contrast to pro-BNP, the association of ICAM levels with cardiovascular events had not been previously described in PLWH. Cell adhesion molecules had been only associated to date with surrogate markers of cardiovascular disease, like the carotid intima-media thickness [43], in PLWH. Soluble CD14 reflects monocyte activation and bacterial translocation, and had also been associated with subclinical atherosclerosis in PLWH [44], but not with ischemic morbidity.

A limitation of the study is the small number of vascular events occurred in the cohort. However, the number of person-years of follow-up was high, older age strata were well represented in spite of the low median age, and the prospective collection from 2008 guarantees the inclusion of the majority of events. Although underreporting cannot be excluded before 2008, the incidence rate was not different or higher than in the period when collection was prospective. The lack of data on cardiovascular risk factors in a high number of participants living with HIV as well as in the general population is another relevant limitation of the study, since this information would significantly strengthen the interpretation of the findings, including the predictive role of the biomarkers. In addition, information on stroke subtypes is not collected in the cohort and, therefore, atherosclerotic cerebrovascular events cannot be distinguished from events caused by other mechanisms for separate analysis. Strengths are the nationwide nature of the cohort, the prospective collection of events provided by each centre, and the cross-checking process to maximize the reliability of data.

In conclusion, the incidence of vascular events in PLWH in Spain is relatively low compared with other Western countries, but rates are higher than in the general population. There is a strikingly low incidence of PAD. The incidence of vascular events increases with age, and they are associated with higher mortality. Compared to the general population, an excess risk of cardiovascular and cerebrovascular events exists at the younger age strata. Serial measurements of biomarkers of inflammation and myocardial function, specifically sICAM and pro-BNP, show increased levels in association with the ulterior development of vascular events in PLWH.

## Supporting information

S1 File Definition criteria for non-AIDS events. (DOCX)

S2 File Dataset. (XLSX)
