# Supplementary material for: Evolving understanding of cardiovascular, cerebrovascular and peripheral arterial disease in people living with HIV and role of novel biomarkers. A study of the Spanish CoRIS cohort, 2004-2015
Source: PLoS One. 2019 Apr 26;14(4):e0215507. doi: 10.1371/journal.pone.0215507 (PMC6485642; doi:10.1371/journal.pone.0215507)
Supplement: S1 File — (DOCX) [file pone.0215507.s001.docx]

**CoRIS**

Fill-in form “NON-AIDS EVENTS” IN CoRIS (NAEs)

| **BASELINE DATA** | | |
| --- | --- | --- |
| **Name of the centre:** | **Date data collection:__/__/__** | **Name of person who fills data:** |
| **Patient’s code:** | **Sex:** | **Date of birth: __/__/__** |

|  |  | **Date of diagnosis** | |
| --- | --- | --- | --- |
|  |  | **Month** | **Year** |
| \| **1. Cardiovascular events** \| \| --- \| | | | |
| a) Coronary: |  |  |  |
| 1) Acute myocardial infarction |  |  |  |
| 2) Angina |  |  |  |
| 3) Sudden death of possible coronary etiology |  |  |  |
| b) Cerebral |  |  |  |
| 1) Transient ischemic attack |  |  |  |
| 2) Reversible ischemic deficit |  |  |  |
| 3) Established stroke |  |  |  |
| 4) Asymptomatic cerebrovascular disease |  |  |  |
| c) Peripheral arterial disease |  |  |  |
| d) Congestive heart failure |  |  |  |
| e) Primary pulmonary hypertension |  |  |  |
| **2. Renal events** | | | |
| a) Acute renal failure |  |  |  |
| b) Chronic kidney disease |  |  |  |
| c) Tubulopathy/Fanconi syndrome |  |  |  |
| d) Symptomatic nephrolithiasis |  |  |  |
| e) Initiation of dialysis |  |  |  |
| f) Kidney transplantation |  |  |  |
| **3. Liver events** | | | |
| a) Hepatic insufficiency/cirrhosis |  |  |  |
| b) Ascites |  |  |  |
| c) Gastrointestinal bleeding by esophageal varices |  |  |  |
| d) Hepatic encephalopathy |  |  |  |
| e) Liver transplantation |  |  |  |
| **4. Neoplastic events** | | | |
| Any neoplasm |  |  |  |
| **5. Bone-related events** | | | |
| a) Vertebral fracture |  |  |  |
| b) Large bone fracture |  |  |  |
| c) Avascular necrosis |  |  |  |
| **6. Neuropsychiatric events** | | | |
| a) Depression |  |  |  |
| b) Suicide attempt/Suicide |  |  |  |
| c) Psychosis |  |  |  |
| **7. Metabolic events** | | | |
| a) Diabetes mellitus |  |  |  |
| b) Lactic acidosis |  |  |  |

**CARDIOVASCULAR EVENT FORM**

**1. Event fill-in form** (please, mark):

** Acute myocardial infarction**

**** Definitive:

1. Diagnostic EKG or

2. Symptoms + probable EKG + rise of cardiac biomarkers (creatin phosphokinase [CK], and MB isoenzyme of CK, LDH, specific troponin T and specífic troponin).

3. Typical symptoms + cardiac biomarkers elevation+ EKG with signs of ischemia, or not codifiable, or not available.

*Diagnostic EKG*: (a) Q wave appearance. If Q wave is equivocal, it must be accompanied by ST or T wave changes. All these changes must be accompanied by progression of T wave in 3 or more derivations; b) evolving ST elevation lasting more than 24 hours and progression of T wave in 3 or more derivations.

*Probable EKG*: a) Non-significant ST drop in a register accompanied by significant drop in another register. b) Non-significant ST elevation in a register accompanied by significant elevation in another register c) Non-significant T wave reversal in one register but significant reversal in another one

Angiography (number of vessels with stenosis):

****Not done

**** 1 vessel

 2 vessels

 3 vessels

>3 vessels

**** Probable

Myocardial infarction characteristics**:**

Transmural (q waves in ECG):

Non-transmural (non q waves)

Killip Classification

****Killip I (no signs/symptoms of left ventricular failure

Killip II (rales or third heart sound or jugular ingurgitation)

Killip III (acute pulmonary edema)

Killip IV (cardiogenic shock)

** Angina** (symptoms suggestive of myocardial ischemia, such as thoracic pain, or pain in the jaw or the arm. Pain usually lasts less than 20 minutes. There must be changes in ECG which conform the existence of myocardial ischemia, such a depression of al least 0.5 mm of ST segment or T wave reversal of al least 1 mm in 2 or more contiguous derivations)

** Sudden death of possible coronary etiology** (typical, atypical or not enough described symptoms and previous history of coronary disease or evidence of coronary disease on autopsy)

** Transient ischemic attack** (Focal neurological deficit due to ischemia of a cerebral territory that lasts less than 24 hours).

** Reversible ischemic deficit** (Focal deficit which lasts more than 24 hours with ulterior reversal).

** Established stroke** (Neurological deficit which does not change during the first 24-72 hours after the initiation).

**** Ischemic

**** Haemorrhagic

**** **Asymptomatic cerebrovascular disease** Patients with vascular risk factors in whom by clinical exam (carotid auscultation), doppler and mainly by neuroimaging studies, have ischemic cerebral asymptomatic lesions (silent infarcts). This group includes also patients with hypodensity in cerebral white matter on CT or MR (leucoaraiosis).

** Peripheral arterial disease**

**** Intermittent claudication

**** Abnormal ankle-brachial index (ABI) (less than 0.9)

**** Other clinical findings (arterial revascularization or previous amputation)

** Congestive heart failure**

**** Class II (NYHA) (mild limitation for ordinary physical activity, such as palpitations or dyspnea, without dyspnea at rest)

**** Class III (NYHA) (marked limitation for ordinary physical activity, without dyspnea at rest)

**** Class IV (NYHA) (dyspnea at rest)

**RENAL EVENT FORM**

**1. Event fill-in form** (please, mark):

** Acute renal failure** was defined as an elevation of creatinine over 1.5 mg/dL, or reaching 1.3 times the upper normal limit value, or a decline in glomerular filtration rate to < 60 ml/min. If creatinine was previously elevated, then an increase higher than 0.5 mg compared to previous value.

** Chronic kidney disease** was defined as either kidney damage or a decreased glomerular filtration rate (GFR) of less than 60 mL/min/1.73 m^2^ for 3 or more months. Kidney damage is defined by anatomopathological changes or by biological markers of kidney damage, including abnormalities in the composition of blood or urine or abnormalities on imaging studies. GFR was estimated using the abbreviated MDRD (Modification of Diet in Renal Disease) equation, and it can be classified in 5 stages according to the glomerular filtration rate (GFR)*** decrease.

*The presence of proteinuria above the maximal diary physiologic excretion (<150 mg/day) is a marker of kidney disease, usually more premature than the GFR decrease. Depending on the quantity of protein excretion, it can be classified in microalbuminuria (30-300 mg/day), non nephrotic proteinuria (300 mg a 3,5 g/day), and nephrotic proteinuria (>3,5 g/day). A result of 1+ or more in reactive labstix reflects a proteinuria of 300-500 mg/day (around 10-30 mg/dL). Persistent proteinuria always reflects renal disease and it can be useful to identify incipient renal disease, in which there is not yet effect on GFR.

**Microhematuria and/or dysmorphic red blood cells and/or cilindruria.

***The quantification of GFR will be calculated with the equations of the MDRD (Modification of Diet in Renal Disease) study:

FGR=186 x (Cr p)^-1.154^ x (age)^-0.203^ x (0.742 if woman) x (1.210 if Afro-American). GFR is expresed in mL/min/1.73 m2, Cr p in mg/dL, anda ge in years.

Stages of chronic kidney disease (please, mark):

**** Kidney damage with GFR>=90 ml/min

**** Proteinuria ****Nephrotic range (> 3.5 g/día)

Non-nephrotic range ****

**** Microhematuria

 Kidney damage with mild decrease of GFR= 60-89 ml/min

 Kidney damage with moderate decrease of GFR = 30-59 ml/min

**** Kidney damage with severe decrease of GFR= 15-29 ml/min

**** Terminal renal insufficiency GFR < 15 ml/min

 Tubulopathy was defined as three of the following:

**** Hypophosphatemia (serum phosphate < 2.7 mg/dL),

**** Proteinuria (at least 1+)

**** Glucosuria (at least 1+ with normal blood gucose),

**** Metabolic acidosis (serum bicarbonate < 23 mEq/L),

**** Hypokalemia (serum potassium < 3 mEq/L),

 Nephrogenic diabetes insipidus,

 Aminociduria

 Hypouricemia

 **Results of renal biopsy** (if performed):

**LIVER EVENT FORM**

**1. Event fill-in form** (please, mark):

** Hepatic insufficiency**: Severe impairment of hepatic synthesis (albumin < 3,5 mg/dl, and/or fibrinogen < 180 mg/dl and/or prothrombin activity < 50% without any other subjacent etiology) and portal hypertension assessed with ultrasonography (splenomegaly, collateral circulation or ascites) or endoscopy (esophageal varices or hypertensive gastropathy) or by direct measure (minimal gradient of hepatic venous pressure of 6 mm Hg), or hepatic encephalopathy history in a patient with chronic liver disease, in the absence of other justifying causes.

** Ascites**: Presence of fluid in peritoneal cavity, observed with image tests (ultrasonography, CT, MR) or confirmed through paracentesis in a patient with known chronic liver disease, in the absence of other justifying causes

** Hepatic encephalopathy**: Mental impairment (usually central nervous system depression) with compatible clinical signs (i.e. asterixis, hyperamonemia, EEG, etc) in a patient with chronic hepatic disease. Other causes of neurologic disease must have been ruled out

**** **Gastrointestinal bleeding by esophageal varices**: Occurrence of hematemesis or melenas with endoscopic evidence of esophageal varices and signs of recent bleeding with

** Hepatic transplant**

**** **Hepatocarcinoma**

**Child-Pugh classification:**

 A (5-6 points)

 B (7-9 points)

 C (10-15 points)


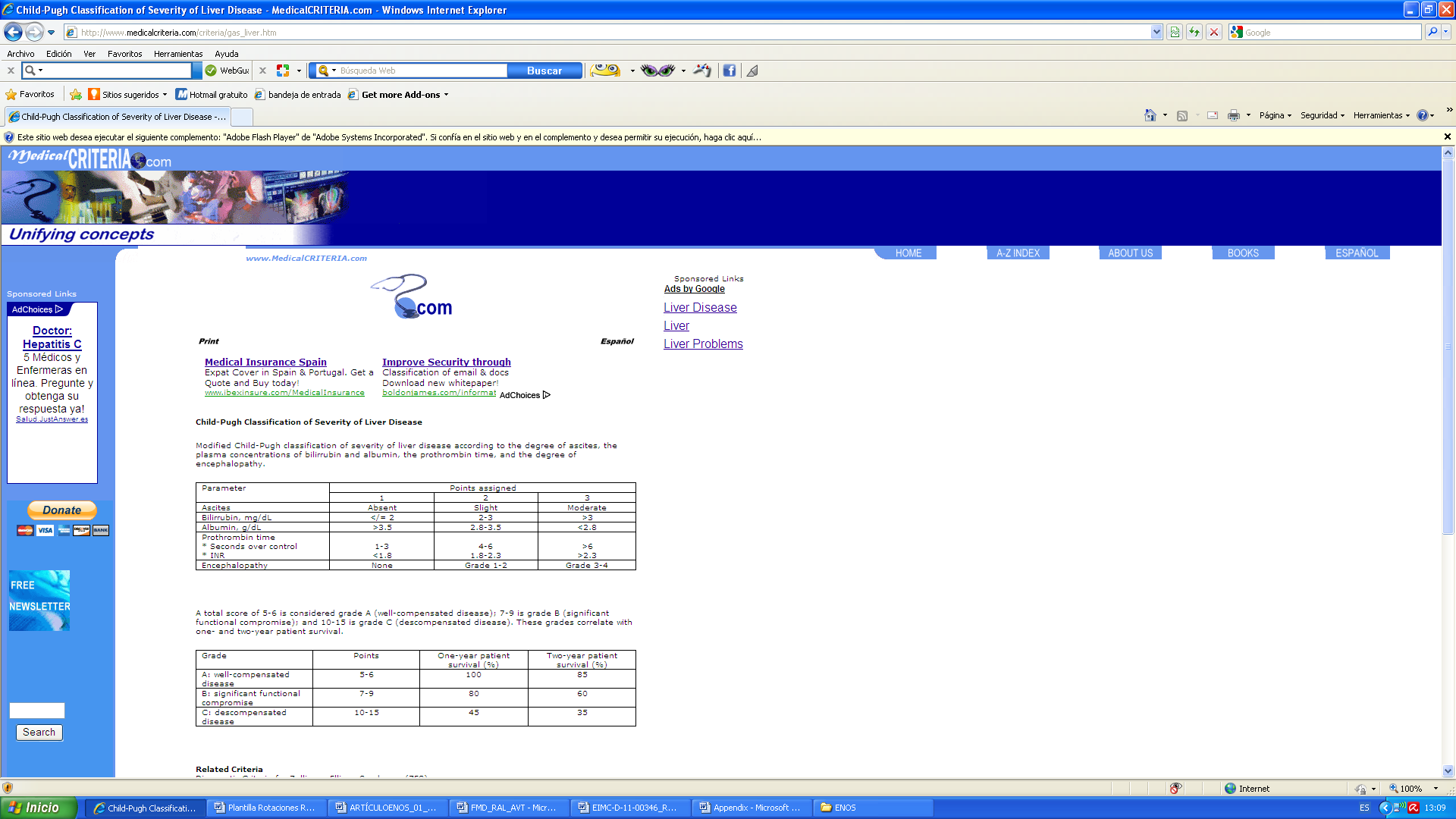


**Image tests**

**** Hepatic space occupying lesions (SOL)

**** No space occupying lesions

**** Unknown

**** Esophageal varices

 No esophageal varices

 Unknown

 Portal hypertension (ultrasonography with splenomegaly or ascites), collateral circulation

 No portal hypertension

 Unknown

**Enolism**

Enolism

No enolism

Unknown

**NEOPLASTIC EVENT FORM**

**1. Event fill-in form** (please, mark):

- Anal cancer
- Rectal cancer
- Bladder cancer
- Prostate cancer
- Breast cancer
- Colonic cancer
- Kidney cancer
- Liver cancer
- Lung cancer
- Stomach cancer
- Uterine cancer
- Head and neck cancer
- Hodgkin lymphoma
- Leukemia
- Melanoma
- Seminoma
- Angiosarcoma
- Brain neoplasm
- Multiple myeloma
- Other

**2. Write the findings associated with this event:**

**Carcinomas**

TNM stage (please, mark):

 M0

 M1

 MX

T1

N0

N1-3

NX

T2

T3

T4

Tis

TX

1. **Primary tumor (T)**

| **TX** | Primary tumor cannot be assessed |
| --- | --- |
| **T0** | No evidence of primary tumor |
| **Tis** | Carcinoma in situ (initial cancer, not disseminated to contiguous tissues) |
| **T1, T2, T3, T4** | Size and/or extension of the primary tumor |

1. **Regional lymphatic nodes (N)**

| **NX** | Not possible to evaluate regional lymphatic nodes |
| --- | --- |
| **N0** | Not complication with regional lymphatic nodes (cancer was not found in regional lymphatic nodes) |
| **N1, N2, N3** | Complication with regional lymphatic nodes (number and/or dissemination extension) |

1. **Distant metastases (M)**

| **MX** | Not possible to evaluate distant metastases |
| --- | --- |
| **M0** | No distant metastases (cancer has not disseminated to other locations in the body) |
| **M1** | Distant metastases (cancer has not disseminated to other locations in the body) |

**Lymphomas**

Stage:

 I: The disease is present in only one group of lymph nodes, or, more rarely, in a single organ that does not belong to the lymph system.

 II: The disease is found in two or more groups of lymph nodes on the same side as the diaphragm. In addition, an organ not in the lymph system may be involved close to the involved nodes.

 III: The disease is present in lymph node groups on both sides of the diaphragm, occasionally with the involvement of other adjacent organs. If the spleen is involved then the disease becomes stage III as well.

 IV: The disease is wide spread, including multiple involvement at one or more extranodal sites (such as the bone marrow).

Grade

 Hodgkin

 Lymphocytic

 Nodular sclerosis

 Mixed cell

 Lymphocytic depletion

**BONE-RELATED EVENT FORM**

**1. Event fill-in form** (please, mark):

**** Vertebral fracture:

**** Localization

**** Severity

**** Grade 1 (20-25%)

 Grade 2 (25-40%)

 Grade 3 (>40%)

 Non vertebral fractures

Localization

**** Hip

 Wrist

 Other***:***

 Avascular necrosis

 Hip

 Shoulder

 Bones of the foot

 Knee

 Wrist

 Other:

Localization:

 Isolated

 Bilateral

 2 areas of different location

 > 2 areas of different location fall

Event description

 Casual drop

 Accident

 Non traumatic

**NEUROPSYCHIATRIC EVENT FORM**

**1. Event fill-in form** (please, mark):

**** Psychosis

**** Schizophrenia

**** Bipolar disorder

**** Psychotic delirium

**** Other:

**** Severe depression that requires pharmacological treatment

**** Suicide/suicide attempt

Predisposing factors

 Toxic dependence

 Others:

**NEUROPSYCHIATRIC EVENT FORM, EXPANDED**

**1. Event fill-in form** (please, mark and describe):

**** Psychosis

**** Schizophrenia

**** Bipolar disorder

**** Psychotic delirium

**** Other (describe):_______________________________________

Clinical description **_______________________________________**

Drugs prescribed **________________________________________**

Outcome_______________________________________________

**** Severe depression that requires pharmacological treatment

Clinical description _______________________________________

Drugs prescribed_________________________________________

Outcome_**______________________________________________**

**** Suicide/suicide attempt

Clinical description _______________________________________

Outcome________________________________________________

**** Predisposing factors

**** Toxic dependence

**** Others (describe):___________________________________________

**METABOLIC EVENT FORM**

| **1. Treatment** |  |  | **3. Findings** |  |  |
| --- | --- | --- | --- | --- | --- |
|  | *Marcar "X"* |  | Plasma lactic acid |  | nn,n mEq/l |
| No therapy |  |  | Plasma bicarbonate |  | nnn mMol/L |
| Diet |  |  | PH |  | n,nn |
| Oral antidiabetics |  |  | Plasma glucose |  | nnnn,n mg/dl |
| Insulin |  |  | AST |  | nnn,n mU/ml para GOT |
| **2. Symptoms** |  |  | ALT |  | nnn,n mU/ml para GPT |
|  | *Marcar "X"* |  | Quick index |  | nnn% |
| Polydipsia |  |  | Other findings: |  |  |
| Polyphagia |  |  |  |  |  |
| Polyuria |  |  |  |  |  |
| Asthenia |  |  |  |  |  |
| Wight loss |  |  |  |  |  |
| Changes in the conscious level |  |  |  |  |  |
| Ophthalmologic disturbances |  |  |  |  |  |
| Other |  |  |  |  |  |
